# Characteristics and health related quality of life in a population with advanced chronic obstructive pulmonary disease, a cross-sectional study

**DOI:** 10.1186/s12904-020-00593-2

**Published:** 2020-06-18

**Authors:** D. G. Bove, M. Lavesen, B. Lindegaard

**Affiliations:** 1 Emergency Department, Copenhagen University Hospital, Nordsjælland, Dyrehavevej 29, 3400 Hillerød, Denmark; 2 Department of Pulmonary & Infectious Diseases, Copenhagen University Hospital, Nordsjælland, Dyrehavevej 29, 3400 Hillerød, Denmark

**Keywords:** COPD, HRQoL, Non-malign palliative care, Patient-reported outcome, Psychosocial characteristics, Quality of life

## Abstract

**Background:**

It is important to understand the total burden of COPD and thereby be able to identify patients who need more intensive palliative care to avoid deteriorated quality of life. The aim of this study was to describe the psychosocial and demographic characteristics of a population with advanced COPD in a stable phase of the disease.

**Methods:**

This study was cross-sectional based on a prospective observational cohort. The following questionnaires were administered: Chronic Respiratory Disease Questionnaire (CRQ), The COPD Assessment Test (CAT), The Hospital and Anxiety and Depression Scale (HADS), The Medical Research Council dyspnoea scale (MRC), and self-rate general health.

**Results:**

We included 242 patients with advanced COPD from a Danish pulmonary outpatient clinic. Their mean FEV_1_ was 38% (±12.7) and 19% were treated with long term oxygen. The mean CRQ domain score was CRQ-dyspnea 4.21 (±1.4), CRQ-Mastery 4.88 (±1.3), CRQ-Emotional 4.81 (±1.2), CRQ-Fatigue 3.93 (±1.3). The mean CAT-score was 18.4 (± 6.7), and 44% had a CAT score > 20. The mean score on the subscale for anxiety (HADS-A) and depression (HADS-D) was 5.07 (±3.9) and 5.77 (±3.9), respectively. Thirty percent self-rated their health as bad or very bad and 19.8% were current smokers.

**Conclusions:**

This study describes the characteristics of a population with advanced COPD in a stable phase of their disease. Our results illustrate how the population although treated in an outpatient structure already focusing on palliative needs, still live with unmet palliative needs and impaired quality of life.

## Background

The World Health Organization (WHO) estimates that 65 million people have moderate to severe chronic obstructive pulmonary disease (COPD) (http://www.who.int/respiratory/copd/burden/en/). In Denmark, approximately 320,000 persons have COPD and 50,000 of these have advanced COPD (https://www.lunge.dk/lunger/viden-noegletal-om-lungesygdomme). Worldwide, 5 % of all deaths are caused by COPD corresponding to more than 3 million annual deaths. In Denmark, 5500 persons die due to COPD each year, making the disease the third most frequent cause of death in Denmark, and the highest mortality rate of COPD in the EU (https://www.lunge.dk/lunger/viden-noegletal-om-lungesygdomme). There is growing evidence that patients with advanced COPD are marked by a high symptom burden, impaired health related quality of life (HRQoL) and live their last years of life with unmet palliative needs [1–5].

In the past years we have developed and implemented a new Danish pulmonary outpatient structure named CAPTAIN with the aim of improving the basic palliative care offered to patients with advanced COPD [6]. All patients were assigned to a CAPTAIN-nurse who were responsible for establishing and maintaining an individualized relationship with the patients and identifying their need for care and treatment. Routine patient controls were replaced with ad hoc physician consultations and planned advance care dialogues. Our qualitative evaluations illustrated how this new outpatient structure made it possible to respond to the individual and fluctuating palliative needs of patients with advanced COPD, and how both patients and health care professionals experienced that the quality of care improved. CAPTAIN is currently considered standard care for all patients with advanced COPD affiliated to our outpatient clinics [6–8].

Until now, the majority of studies investigating symptom burden and HRQoL in patients with COPD include mainly patients with COPD in a mild or moderate state and treated in traditional organizations with semi-annual or annual medical checks [9, 10]. The explanation may be that it is difficult to recruit patients with advanced COPD to research projects, just like it requires special attention and devoted resources from the researchers to reduce missing data and dropouts. The CAPTAIN structure and assigned CAPTAIN-nurses gave us the opportunity to collect this data through validated questionnaires - either administered by self-report or interview, and thereby generate knowledge about the population with advanced COPD in a stable phase of the disease.

There is a need for knowledge about the symptom burden and HRQoL in patients with advanced COPD, living at home and treated in an outpatient structure that already focuses on the patients’ individual basic palliative needs. HRQoL is arguable the most relevant outcome from a patient’s perspective and associated with both COPD mortality and morbidity [9, 11–14]. Therefore, the aim of this study was to describe the psychosocial and demographic characteristics of a population with advanced COPD in a stable phase of the disease.

## Methods

### Study design

This study was cross-sectional based on a prospective cohort of patients with advanced COPD affiliated a pulmonary outpatient clinic at Nordsjællands Hospital, Denmark. In Denmark, only patients with advanced COPD are seen in a pulmonary outpatient clinic, otherwise they are cared for by their general practitioner. Advanced COPD was defined as a FEV_1_ less than 50% of predicted or a high symptom burden or two or more annual exacerbations [5]. CAPTAIN was standard care for all patients [6].

### Study population

All patients affiliated or referred to the pulmonary outpatient clinic with the diagnosis COPD (ICD-10) could be included (*n* = 650). Exclusion criteria was inability to understand Danish or unwillingness to give informed consent.

Ten patients did not meet the inclusion criteria, 28 patients rejected to participate due to lack of resources, poor vision or fatigue and 50 patients verbally agreed to participate but were never included as they did not fulfill or return the informed consent. Three hundred twenty patients were not invited to participate by their nurse. The nurses’ reason for this was either bustle and lack of time among the nurses, or because the nurses interpreted that the patients were in a too bad condition to participate or in acute phase of the disease.

A total of 242 patients with advanced COPD were enrolled in the period June 2017 to December 2018.

### Data colleting and data management

The first and second author reviewed the patients’ medicinal records for demographic and clinical data (Table 1). Weight, FEV_1_ and oxygen saturation were measured by inclusion. If this was not possible, the last measured values within the previous 12 months were recorded. Data about smoking and alcohol habits were patient reported at time of inclusion.

**Table 1:** Type of variables

Characteristics	Type of quantity
***Sociodemographic***	
Age, height, weight	Continuous
Gender, ethnicity, marital, educational and occupational status, living arrangements	Categorical
Use of social services, smoking, alcohol, E-cigarettes	Binary (Y/N)
***Clinical and paraclinical***	
FEV_1_ of predicted value, oxygen saturation, numbers of exacerbation within 12 months, BMI	Continuous
Oxygen treatment, treated with NIV within 12 months,	Binary (Y/N)
***Medications***	
SSRI, TCA, azapirones	Binary (Y/N)
Benzodiazepines	Binary (Y/N)
Opioids on the indication dyspnoea	Binary (Y/N)
Permanent corticosteroid treatment	Binary (Y/N)
Weak analgesics and nonsteroidal anti-inflammatory agents	Binary (Y/N)
LAMA, LABA, ICS or combinations	Binary (Y/N)
Cannabis oil	Binary (Y/N)
***Usual care***	
Pulmonary rehabilitation during the past 12 months	Binary (Y/N)
Practical assistance, personal care, home nursing care	Binary (Y/N)
Public appropriation for terminal care	Binary (Y/N)
***Mortality***	
Death	Binary (Y/n)
Place of death	Categorical
***Standardised questionnaires***	
CRQ, CAT, HADS, MRC	Continuous
Self-rated health	Categorical

*FEV1* forced expiratory volume in 1 s; *BMI* body mass index; *NIV* non-invasive ventilation; *SSRI* selective serotonin reuptake inhibitors; *TCA* tricyclic antidepressants; *MRC* Medical Research Council dyspnoea scale; *CAT* The COPD Assessment Test (CAT); *CRQ* Chronic Respiratory Disease Questionnaire; *HADS* Hospital and Anxiety and Depression Scale; *Y/N* yes/no.

Patients completed self-reported or interview administrated questionnaires. All included patients were asked to fill out the following questionnaires: Chronic Respiratory Disease Questionnaire (CRQ), The COPD Assessment Test (CAT), The Hospital and Anxiety and Depression Scale (HASDS), The Medical Research Council dyspnoea scale (MRC), and in addition self-rate their general health. Licenses were obtained on all questionnaires before the start of the study and the standardised questionnaires were scored according to the guidelines from the instrument developers.

All data were entered into a RedCap database by DGB and ML and two independent student workers. Finally, the two student workers controlled 20% of all entered data and found minor data entry errors in three cases.

### Measurements

The CRQ measure HRQoL in patients with respiratory diseases. We used the self-administrated standardized CRQ (CRQ-SAS) which consist of 20 items across four dimensions; Dyspnea (5 items), fatigue (4 items), emotions (7 items) and mastery (4 items). Patients answer each question on a seven points Likert-type scale to express the degree of disability from 1 (maximum impairment) to 7 (no impairment). The mean score of each domain is calculated and presented for interpretation (range 1–7). It is not recommended to present a summary score of the 20 items [15].

The CAT is a self-administered questionnaire that measures health status in patients with COPD. The CAT consists of 8 items (cough, phlegm tightness, breathlessness, limited activities, confidence leaving home, sleeplessness and energy) each assessing the impact on COPD on daily life and rated on a semantic differential scale from 0 to 5. The total CAT score ranges between 0 (low impact) to 40 (high impact) [16]. A CAT-score between 10 and 20 points is defined as of medium impact on patients’ HRQoL and COPD described as the most important problem patients have. A CAT-score > 20 points are described as COPD stops patients from doing most things that they want to do and with high impact on their HRQoL (http://www.catestonline.org/images/UserGuides/CATHCPUser%20guideEn.pdf).

The HADS is a self-completed questionnaire that measure symptoms of anxiety and depression in patients in non-psychiatric settings [17]. HADS consist of two subscales, where anxiety (HADS-A) and depression (HADS-D) are assessed as separate components each with seven items rated on a four-point scale from 0 (no symptoms present) to 3 (significant symptoms). The score on each subscale range from 0 to 21. A score above 8 points on each subscale indicates clinically significant symptoms of anxiety and/or depression [18].

The MRC is a patient-rated single item scale where severity of dyspnea is rated by the patient from 1 to 5. ‘I only get breathless with strenuous exertion’ is grade 1 and ‘I am too breathless to leave the house’ is grade 5. An MRC ≥ 3 is a threshold for separating less breathlessness from more breathlessness [5, 19].

### Statistical methods

All variables were analysed in a descriptive manner (Table 1). Parametric data were analyzed using means, standard deviations (SD) and ranges, and nonparametric data with medians and interquartile ranges (IQR). Comparison between means was conducted with t-tests. The association between mortality (outcome) and the four instruments (CRQ, HADS, CAT, and MRC) (exposures) was tested using a multivariable logistic regression analysis with gender and age adjustment. Statically significant results were defined as those with *p* < 0.05. Statistical analysis was performed using SPSS v.22 (IBM Corp., Armonk, NY, USA).

There are two main types of missing data; missing forms or missing items. In missing forms, the whole questionnaire is missing, while missing items refer to one or several items in the questionnaire is not completed. In this study, all questionnaires not 100% completed are excluded from the analysis, which is why the numbers (n) varies in Tables 1 and 3.

## Results

Demographic and clinical baseline characteristics of the included patients (*n* = 242) are presented in Table 2. The patients were aged 39–91 years with a mean of 72 years (8.4) and 58% were females. Except for one patient, all had an ethnicity of Danish origin. One fifth of the patients were current smokers and more than 13% consumed more alcohol than recommended by the Danish National Board of Health.

**Table 2:** Demographic and clinical characteristics of the patients (*n* = 242)

***Characteristics***	***N (%)***	***Mean ± SD***	***Range***
Gender, females	140 (57.9)		
Age, year		72.01 ± 8.40	39–91
Ethnicity, Danish origin	241 (99.6)		
Body Mass Index (kg/m^2^)		25.11 ± 6.20	11–44
***Smoking and alcohol consumption***			
Current smoker	48 (19.8)		
Current E-cigarette user	7 (2.9)		
More than 7 units of alcohol per week for women and 14 units for men	33 (13.6)		
***Living arrangements***			
Living alone	102 (42.1)		
Nursing home resident	3 (1.2)		
***Educational and occupational status***			
No education	16 (6.6)		
Medium academic or trade	129 (53.3)		
Academic	21 (8.7)		
Retired due to health or age	215 (88.8)		
Employed	18 (7.4)		
***Social network***			
The presence of a well-functioning and solid network, self-rated	225 (93.0)		
***Use of social services delivered by the municipality***			
Help with personal care	36 (14.9)		
Help solving practical tasks	57 (23.6)		
Primary nurse care	30 (12.4)		
***Pulmonary function and oxygen therapy***			
Long term oxygen therapy	46 (19.0)		
FEV_1_% of predicted value		38.04 ± 12.74	10–77
Saturation O2		94.54 ± 2.28	84–100
***Non-Invasive Ventilation (NIV) the previous 12 months***^***a***^***(n = 229¤)***			
NIV	27 (11.8)		
***Exacerbations treated with systemic corticosteroids, antibiotics, or both the previous 12 months (n = 237¤)***			
No exacerbations	81 (34.2)		
1 or 2 exacerbations	107 (45.14)		
More than 2 exacerbations	49 (20.74)		
***Pulmonary rehabilitation***^***b***^***the previous 6 months***^***a***^			
*Participated*^*c*^in pulmonary rehabilitation*	58 (24.0)		

*Results expressed are numbers (n), Percentages (%), means with standard deviations (SD) and ranges.*

^*a*^*Previous 6 or 12 months refers to the time from baseline and 6 or 12 months back in time*

^*b*^*Pulmonary rehabilitation includes physical training combined with patient education and has a duration of 10 weeks.*^*c*^*Participating is defined as at least 50% attendance. ¤The actual number included in the analysis*

Patients demonstrated a mean FEV1 of 38% of predicted value (12.7), and 19% were treated in their home with long term oxygen. In addition, 12% of the patients had been treated with NIV in the previous 12 months and 66% (the sum of patients with 1 or 2 exacerbations and those with > 2 exacerbations) of the patients had one or more exacerbations within the last 12 months.

Selected pharmacological preparations extracted from the patient’s record is presented in Table 3. Nearly 50 % of the patients were treated for pain (weak analgesics, NSAID or opioids). Thirteen patients (6%) were treated with opioids to relieve their dyspnea, whereas only one patient was treated with cannabis oil. Thirty-two patients (13%) were treated with antidepressant, ten (4%) with antipsychotics and six-teen (7%) with benzodiazepines.

**Table 3:** Concomitant medication

Pharmacological variables (***n*** = 242)	n (%)
***Regularly orally administered medication***	
Weak analgesics and nonsteroidal anti-inflammatory agents (NSAID)	82 (33.9)
Opioids on indication pain	29 (12.0)
Opioids on indication dyspnea	13 (5.4)
Benzodiazepines	16 (6.6)
Antidepressants	32 (13.2)
Antipsychotics	10 (4.1)
Cannabis oil	1 (0.4)
***Regularly inhaled medication***	
Short acting bronchodilaters	219 (90.5)
LAMA^a^	3 (1.2)
LABA^b^	3 (1.2)
ICS^c^	1 (0.4)
LAMA + LABA	85 (35.1)
ICS + LABA	8 (3.3)
ICS + LAMA	2 (0.8)
ICS + LABA + LAMA	138 (57.0)

^a^*LAMA* long-acting muscarinic antagonist; ^b^*LABA* long-acting β2-agonist; ^c^ICS, inhaled corticosteroid

More than 50 % of the patients were prescribed triple therapy (ICS/LABA/LAMA) and the rest a two-drug combination. Only seven patients received single drug treatment. Not surprisingly, more than 90 % were prescribed short acting bronchodilators.

Table 4 shows the patient reported psychosocial outcomes assessed by the standardised questionnaires. The CRQ assessed HRQoL based on four domains. Each domain was scored separately, and higher scores indicated better quality of life. On average did the patients have a CRQ-D score of 4.21 points, CRQ-M of 4.88 points, CRQ-E of 4.81 points and CRQ-F of 3.93 points. The patients had on average the lowest score on the fatigue domain indicating most HRQoL impairment compared to the domains: dyspnea, mastery and emotion. However, all patients experienced impairment in all domains.

**Table 4:** Patient-reported psychosocial variables assessed by the questionnaires CRQ. HADS, CAT, MRC and self-rated health

Questionnaires	Mean ± SD	N (%)	Range
***Quality of life according to CRQ*** (*n* = 201^a^)			
CRQ-D	4.21 ± 1.44		1–7
CRQ-M	4.88 ± 1.32		1–7
CRQ-E	4.81 ± 1.24		1–7
CRQ-F	3.93 ± 1.30		1–7
***Depression and anxiety according to HADS*** (*n* = 194^a^)			
HADS-A	5.07 ± 3.92		0–17
HADS-D	5.77 ± 3.89		0–19
***Quality of life according to CAT*** (*n* = 225^a^)			
CAT	18.41 ± 6.68		2–38
***Functional dyspnoea according to MRC*** (*n* = 209^a^)			
MRC	3.35 ± 1.12		1–5
***Self-rated health*** (*n* = 183^a^)			
Very good		12 (6.6)	
Good		22 (12.0)	
reasonable		93 (50.8)	
Bad		45 (24.6)	
Very bad		11 (6.0)	

CRQ domain scores with values from 1 (largest impairment) to 7 (no impairment). Each HADS subscale scores with values from 0 to 21 (high score corresponds to level of symptoms). CAT scores from 0 to 20 (high scores correspond to level of impairment). MRC scores from 1 to 5 (high score correspond to high intensity of dyspnea). ^a^The actual number included in the analysis.

The mean CAT-score was 18.41 points. Forty-four percent had a CAT score ≥ 20 points and more than 90% a score ≥ 10 points. The mean MRC was 3.35 points and 73% had an MRC score ≥ 3.

Sixty patients (32%) had a HADS-D subscale score ≥ 8 point and forty-five patients (24%) a HADS-A subscale score ≥ 8 points. Thirteen percent of the patients had a both HADS A and a HADS D score ≥ 8 point.

More than half of the patients rated their health as reasonable and 30% as bad or very bad. However, 18.5% rated their health as very good or good.

Twenty-nine patients died during the data collection period of 1.5 year corresponding to a crude mortality rate of 12%. The majority, 70% died in the hospital, 24% died at home and 6% died at hospice. The patients who died consisted of 58.6% males, and 66% were living alone. On average 31% of the patients who died rated their health as bad or very bad. Their mean CRQ-domain scores were: CRQ-D 3.7 (±1.4), CRQ-F 3.4 (±1.4), CRQ-E 4.5 (±1.4), CRQ-M 4.6 (±1.5). Their mean CAT score was 18.54 (±4.6). They had a mean HADS-A score of 6.6 points (± 5.3) and a HADS-D score of 7.3 (± 5.1). The patients (*n* = 29) who died had an average of 1.52 points [95% CI, 0.10 to 2.94] higher HADS-D score compare to the rest of the patients (*p* = 0.0454). On the HADS-A, the difference was on average 1.51 points [95% CI, 0.07 to 2.95] higher score (*p* = 0.049). The frequency of ≥2 annual exacerbations were 41.4% in the patients who died compared to 34.1% among the rest of the patients. A multi logistic regression analysis did not show any significant association between mortality and CAT, MRC, CRQ, and HADS scores with and without adjustments for age and gender (data not shown). There was a significant association between age and mortality OR: 1.09, [1.001; 1.18] *p* = 0.05.

### Data completeness of administrated questionnaires

The overall data quality in terms of completed and returned questionnaires was acceptable, as all questionnaires expect self-rated health had a completeness > 80%. The completeness ranged between 76% for self-rated health to 93% for the CAT. Missing data present a serious problem in any study, and it is always an issue whether those who completed the questionnaires differ from those missing. Based on rule of thumps missing data between 5 and 20% is acceptable and considered expected and realistic in a population marked by severe disease [20–22].

## Discussion

Our study contributes knowledge of the psychosocial status of patients with advanced COPD in a stable phase of the disease and show a widespread level of HRQoL impairment. Nearly all patients had a CAT score ≥ 10 points and more than 44% a CAT-score ≥ 20 points. The Global Initiative for Chronic Obstructive Lung Disease (GOLD) recommend a CAT score ≥ 10 points as a threefold for considering treatment for symptoms [5] which comply with the recommendations of offering all patients with advanced COPD early integrated palliative care [11, 23, 24].

Although the patients in our study were treated in an altered outpatient structure focusing on the patients’ basic palliative needs [6, 7], our patients still lived with impaired HRQoL. However, we cannot know if our population’s HRQoL actually were higher compared to patients treated in traditional outpatient clinics. Nibber et al. found in a retrospective cohort of patients with severe/very severe COPD a mean (SD) CAT-score of 26.1 (7.6) points [25], which is significantly higher than our mean CAT-score of 18.41 (6.7) points.

According to the CRQ was fatigue the problem affecting the patients’ HRQoL most. Fatigue is a cardinal but multifaceted symptom of advanced COPD and described as a profound feeling of physical and psychological wariness that is not relieved by sleep or rest [11, 26]. Studies report fatigue prevalences in patients with advanced COPD to 71 and 96% in palliative patients [26]. Fatigue do often co-exist with breathlessness and cough in what is called the respiratory cluster [11, 27], just like fatigue is associated with depression and anxiety [28].

In this study the prevalences of anxiety and depression were within the ranges of what is previous described for COPD outpatients in a stable phase of the disease [28]. However, it is worth noticing that 13% of the patients did suffer both symptoms of anxiety and depression; a known condition with heightened risk of suicidal ideation, increased physical disability and chronic depressive symptoms [29, 30]. The frequency of patients treated with benzodiazepines in this study were lower than reported by Vozoris et al. in a population of older adults with COPD [31]. Nearly 50 % of patients in this study were treated for pain which correspond to the findings of others reporting prevalences of pain ranging from 32 to 77% in patients with all stage COPD [32, 33]. However, little is known about the cause and level of pain in patients with advanced COPD and how pain is effectively managed both pharmacological and non-pharmacological in this population [33]. It can be assumed that the cause of pain in patients with advanced COPD can be muscular, related to osteoporosis, arthritis or other comorbidities just like pain properly will be associated with anxiety, depression and dyspnea. Further research is recommended.

The patients who died (*n* = 29) had a clinically significant higher level of anxiety and depression compared to the rest of the patients [17], although it did not show in impaired HRQoL assessed by the CRQ or CAT. In this study, the prevalence of two or more annual exacerbations were more frequent among the patients who died compared to the rest of the patients. This result is not surprising as it is well known that COPD patients with comorbid anxiety and/or depression have a higher risk of exacerbations and mortality after exacerbations, compared to patients without these comorbidities [12, 34, 35]. The majority of the patients died in the hospital, which mirror other studies results, although the patients’ general preference is to die at home [11, 36–38].

It is thought-provoking that despite the population was characterized by severe pulmonary disease, one-fifth was current smokers. Although patients with advanced COPD are in the last stage of the disease and presumably have a limited life span, smoking cessation still makes sense and contribute to prevent further deterioration [5, 39]. In Denmark, smoking cessation courses are free but requires attendance at a given location outside the patients’ home. Transport are a known barrier for patients with advanced COPD and a significant reason that they do not profit from health promotion intervention outside their home [40, 41]. It would have been interesting if we had collected data on whether smoking patients had been offered smoking cessation interventions in form of pharmacology, non-pharmacological or combinations. Our results underpin a need for further knowledge about how to motivate and support patients with advanced COPD with smoking cessation.

With this study we have described the characteristics of a population with advanced COPD. The prevalences of high CAT, MRC, HADS, or low CRQ scores were high, and indicate a need for a more intensive palliative care intervention than actually offered. Intensive palliative care should be individualized and address the needs that are currently most relevant to the patient concerned. Systematic screening of the symptom burden using validated questionnaires could be a method of targeting and intensifying a palliative effort.

Our results illustrate that if we for example define a CAT-score above 20 points as an indicator for intensified palliative needs [5], the target population will be huge and at the same time not target the patients who die as they had a mean CAT score of 18 points. In Denmark 50,000 patients suffer from severe COPD [2]. It may be assumed that many of them have a CAT score above 20 points, and if 44% of these patients were offered intensified palliative care this alone would correspond to 22,000 patients. In addition, our population may not be fully representative for the general population of patients with severe COPD. Our patients are already treated in a palliative care setting [6], and the percentages with a CAT scores above 20 points may therefore be even higher than the 44% in a general COPD population.

In Denmark, specialized palliative care is initially developed and targeted to cancer patients and organized in oncology. Currently, the need for specialized palliation and hospice stays among Danish cancer patients exceeds the current capacity and patients with cancer are often waitlisted before receiving a palliative offer (www.repha.dk). More than 95 % of the specialized palliative resources in Denmark are used on cancer patients, and only few COPD patients are offered specialized palliative care or admitted to a hospice [42–45]. The size of the COPD population with unmet palliative needs makes it, in our opinion, unrealistic to think that patients with advanced COPD can be accommodated into existing specialized palliative offers. We recommend that future research and initiatives aiming at improving palliative care for COPD patients take the COPD population size into consideration. If we want to offer early and integrated palliative care and be able to accommodate the size of the COPD population, we have to reflect on how to use and expand our existing outpatient structures and competences among pulmonary nurses and physicians in a formalized collaboration with the specialized palliation.

### Limitations and strengths of this study

This study has several limitations. The exact numbers and reason for not participating are not registered systemically, and we cannot know if the patients included are representative for the population with advanced COPD. It can be assumed that the patients included in this study and willing to complete several questionnaires are those who are least affected by their disease and thereby those with the best quality of life. We see the following strengths of our study. First, it was possible for us to include patients with advanced COPD. Second, our study sample was rather large compared to other studies investigating the symptom burden and quality of life in patients with advanced COPD [46, 47]. Third, our patients were treated in an described outpatient structure based on existing guidelines for patients with advanced COPD [6–8]. Fourth, we only used validated questionnaires [14, 18, 48–51].

## Conclusion

This study describes the characteristics of a population with advanced COPD in a stable phase of the disease. If a CAT-score above 20 points are used as an indicator for intensified palliative needs in patients with COPD, the target population will be huge and at the same time not target the patients who die. Our results illustrate how the population, although treated in an outpatient structure already focusing on palliative needs, still live with unmet palliative needs and impaired HRQoL and how one out of five patients currently smokes.

## Data Availability

The datasets analysed during this study are available from the corresponding author on reasonable request.

## Abbreviations

BMI: Body Mass Index
CAT: The COPD Assessment Test
CRQ: The Chronic Respiratory Disease Questionnaire
COPD: Chronic Obstructive Pulmonary Disease
FEV_1_: Forced Expiratory Volume in 1 s
HADS: The Hospital and Anxiety and Depression Scale
HRQoL: Health Related Quality of Life
MRC: The Medical Research Council Dyspnoea Scale
NIV: Non-invasive ventilation
NSAID: Nonsteroid Anti-inflammatory Agents
SSRI: Selective Serotonin Reuptake inhibitors
TCA: Tricyclic Antidepressant
WHO: World Health Organization
